# Case Report: Metabolic adaptations to intensive aerobic training in Parkinson's disease: autonomic and physiopathological response

**DOI:** 10.3389/fspor.2026.1920699

**Published:** 2026-10-07

**Authors:** Annalisa D’Ermo, Rossella Rotondo, Tommaso Di Libero, Giada D’Alessandro, Angelo Rodio, Elvira Padua, Fabrizio Stocchi, Michela Goffredo, Michele Guescini, Vilberto Stocchi, Giuseppe Rosano, Maria Francesca De Pandis

**Affiliations:** 1 Department of Human, Social and Health Sciences, University of Cassino and Southern Lazio, Sustainable Living Concept Laboratory “Marco Marchetti” (Xlab), Campus Folcara, Cassino, Italy; 2 IRCCS San Raffaele Roma, Cassino, Italy; 3 Department of Human Science and Promotion of Quality of Life, San Raffaele Rome Open University, Rome, Italy; 4 European University of Technology, EUt+ (University of Cassino and Southern Lazio), Cassino, Italy; 5 IRCSS San Raffaele Roma, Rome, Italy; 6 Department of Biomolecular Sciences, University of Urbino Carlo Bo, Urbino, Italy

**Keywords:** aerobic training, autonomic dysfunctions, case report, METs, Parkinson's disease, physical exercise, rehabilitation

## Abstract

**Introduction:**

Aerobic exercise is a key non-pharmacological intervention in Parkinson's disease (PD), yet the physiological mechanisms underlying its therapeutic effects remain incompletely understood. This case report aimed to investigate metabolic and cardiovascular adaptations to an intensive aerobic training programme in a person with PD, with particular focus on metabolic efficiency and insulin-like growth factor 1 (IGF-1) response.

**Methods:**

A 72-year-old man with idiopathic PD (Hoehn & Yahr stage II, ON medication) participated in a 4-week intensive treadmill training programme (20 sessions) within the METEX-PD protocol. Exercise was performed at a constant external workload (5.0–5.5 km·h⁻^1^; 6.0–8.8 METs). Heart rate (HR) was continuously monitored, oxygen uptake ($\dot{V}$O₂) was assessed using a portable metabolic analyser, post-exercise blood lactate concentration was measured, and serum IGF-1 levels were evaluated. Assessments were conducted at baseline, mid-intervention, and post-training.

**Results:**

Progressive reductions in HR, $\dot{V}$O₂, measured METs, and energy cost were observed throughout the intervention, indicating improved aerobic efficiency despite the maintenance of a constant external workload. HR responses across training sessions showed a peculiar pattern suggestive of chronotropic incompetence and cardiovascular drift. Non metabolic Hyperventilation occurred during post training testing. Serum IGF-1 levels increased following the training period, whereas no acute exercise-induced changes were detected. Blood lactate responses remained consistent with the prescribed exercise intensity.

**Discussion:**

Intensive aerobic training induced favourable metabolic adaptations in this individual with PD, as evidenced by improved aerobic efficiency and increased circulating IGF-1 levels. These adaptations occurred despite potential autonomic dysfunction affecting cardiovascular responses. The findings support the use of direct metabolic measurements to better characterise training adaptations and optimise individualised aerobic exercise prescription in people with PD. Further studies are warranted to confirm these observations in larger cohorts.

## 1 Introduction

Parkinson's disease (PD) is a progressive neurodegenerative disorder characterized by motor symptoms, as well as by many non-motor features (1) that have an impact on disability and quality of life (2, 3).

Over the past decades, physical exercise, particularly aerobic training, has emerged as a potential “universal prescription” for PD, showing beneficial effects on both motor and non-motor symptoms, slowing functional decline, and possibly promoting neuroplasticity in people with PD (4).

Multiple randomized controlled trials and meta-analyses have shown that aerobic exercise leads to significant improvements in motor function, as measured by MDS-UPDRS Part III scores, and enhances quality of life in people living with PD (4).

Furthermore, several studies have shown that physical exercise positively affects non-motor symptoms, such as cognitive function (5, 6) and neuroprotective effects (7), mood disorders (8) and sleep quality (9), contributing to an overall improvement in patients’ clinical status (10, 11). Despite these promising results, the optimal “dose” of exercise, defined by the combination of intensity, frequency, duration, and type, remains uncertain, especially in terms of maximizing clinical benefit and inducing neuroplastic adaptations.

To address this gap, the METEX-PD protocol (12) (no Protocol 0107243/2023, registered on ClinicalTrials.gov with the identification number: NCT06339398) was developed to translate and adapt the principles outlined in the 2018 US Physical Activity Guidelines and the WHO Guidelines on Physical Activity and Sedentary Behavior (13) to the specific needs of individuals with PD (14). These international guidelines recommend that healthy older adults (≥65 years) engage in 150–300 minutes per week of moderate-intensity aerobic activity (3.0–5.9 METs) or 75–150 minutes per week of vigorous-intensity activity (≥6.0 METs). In these guidelines, exercise intensity is expressed in metabolic equivalents of task (METs), and duration in minutes per week, providing a quantitative framework for prescribing and monitoring aerobic exercise (15).

The METEX-PD study applies this model by standardizing aerobic exercise volume through the metric of MET-minutes per week, calculated as the product of exercise intensity (in METs) and duration (in minutes). This approach to exercise quantification was adopted with the aim of monitoring energy expenditure and potentially facilitating comparisons between exercise regimens across participants and clinical settings. Participants are assigned to either a low-volume (moderate-intensity) or high-volume (vigorous-intensity) aerobic training group, with the aim of determining whether a higher exercise dose yields greater clinical and biological benefits, including the upregulation of neurotrophic factors and improvements in motor function, cognition, gait, and brain connectivity.

The present case report therefore aims to provide a detailed mechanistic characterization of the early cardiorespiratory, metabolic and biological adjustments and adaptations occurring during protocol implementation, generating hypotheses that may inform the interpretation of the future cohort results.

Ultimately, to be effective, exercise must be prescribed with the same precision as pharmacological treatments. In this context, volume can be expressed using parameters such as time, repetitions, distance, steps, or kilocalories, while intensity can be quantified through physiological parameters including heart rate (HR), blood lactate, and metabolic/ventilatory measures such as oxygen uptake ($\dot{V}$₂) and carbon dioxide production ($\dot{V}$CO₂). The study of cardiac activity is the easier used parameter to monitor exercise intensity in clinical trials for people with PD expressed as absolute value, percentage of maximum HR (%HRmax) or HR reserve (%HRR) (14). Its popularity is also due to the widespread use of wearable devices, which allow easy self-monitoring without specific electromedical devices or medical supervision.

While the availability of multiple monitorable parameters represents a clear advantage, as it allows interventions to be adapted to the resources at hand, it simultaneously complicates the standardization of exercise-based treatment protocols.

Indeed, several reviews have highlighted substantial heterogeneity in exercise prescription and testing (16–18) modalities in Parkinson's disease, including variability in intensity markers (HR, VO₂, RPE), exercise modalities, and progression criteria. This heterogeneity limits the comparability of findings across studies and hampers the translation of evidence into clinical practice.

In this context, our study aimed to improve standardization and transparency of exercise dose by: explicitly defining external workload parameters (e.g., walking speed and treadmill inclination), quantifying exercise dose using METs, and prescribing weekly exercise volume based on MET accumulation, in line with World Health Organization physical activity recommendations.

Moreover, within the METEX-PD trial framework, this case study explores the cardiovascular and respiratory adaptations to continuous, constant-load aerobic exercise in a person with Parkinson's disease, drawing parallels with physiological responses observed in athletic training. Given the presence of autonomic dysfunction in Parkinson's disease, phenomena such as Chronotropic Incompetence(CI) (19, 20), may be present, while during prolonged steady-state exercise Cardiovascular Drift (CVD) (21, 22), may also occur. In this context, by monitoring heart rate (HR) and gas exchange parameters (O₂ and CO₂) during exercise, the study aims to characterize the individual's cardiorespiratory response and adaptation to sustained aerobic effort, while also exploring the occurrence and potential physiological determinants of these responses. Specifically, we assess cardiovascular function via HR, quantify exercise intensity through gas exchange analysis, and evaluate metabolic recovery by measuring post-exercise blood lactate concentration in a person with Parkinson's disease. By integrating direct metabolic measurements with cardiovascular and biological markers, this case report seeks to provide mechanistic insights into acute and chronic changing induced by exercise that may not be readily identifiable from group-level analyses alone.

### 1.2 Participant

To be eligible for inclusion in the study, participants were required to meet the following criteria, as outlined in our previously published study protocol (approved by the local Ethics Committee, protocol no. 0107243/2023, and registered on ClinicalTrials.gov: NCT06339398) (12): (i) a diagnosis of PD based on the UK Parkinson's Disease Society Brain Bank criteria (23); (ii) age between 30 and 80 years; (iii) disease stage II or III in the “ON” medication phase, according to the modified Hoehn and Yahr scale (24); (iv) no evidence of severe cognitive impairment, defined as a Mini-Mental State Examination (MMSE) score ≥24 (25) and a Montreal Cognitive Assessment (MoCA) score ≥17 (26); (v) a stable dopaminergic treatment regimen for at least four weeks prior to enrolment; (vi) physical ability to perform the 6-Minute Walking Test (6MWT); and (vii) willingness and ability to participate in the study, understanding the study procedures and providing written informed consent. Following routine clinical practice, the medical history and clinical status of patients with PD, including cardiological examination, neurological assessment, routine laboratory analyses, and neuropsychological evaluation, were documented upon admission to either the intensive or non-intensive rehabilitation program. As part of the standard screening procedures, autonomic function was assessed to ensure hemodynamic stability during the exercise program and to minimize potential confounding factors in the interpretation of cardiovascular and respiratory responses.

Table 1 summarizes the demographic characteristics, anthropometric measurements, vital signs, respiratory parameters, medical history, neurological and neuropsychological evaluations, as well as laboratory findings collected during the screening visit.

**Table 1:** Summary of demographic, clinical, and functional assessments at screening visit.

Clinical parameters	Variables	
Demographic data	Sex, (male/female)	Male
	Age (years)	72
	Education (years)	18
	Ethnicity	Caucasian
Anthropometric data	Height (cm)	169
	Weight (kg)	73
	BMI (kg/m^2^)	25.56
Vital signs	Systolic Blood Pressure (mmHg)- lied down	140
	Diastolic Blood Pressure (mmHg) – lied down	80
	Heart Rate (beats/minute) – lied down	60
	Systolic Blood Pressure (mmHg)- Standing	138
	Diastolic Blood Pressure (mmHg) - Standing	77
	Heart Rate (beats/minute)- Standing	63
Physical fitness level 6 MWT	Travelled Distance	550 m
	Speed	1.53 m/sec
Medical history	Disease Duration	5
	Age at onset	67
	Comorbidities	None
Antiparkinsonian drugs	LEDD (mg)	520
H&Y (ON phase)	Stage	2
MDS – UPDRS I	Phase ON, 0–52 points	9
MDS - UPDRS II	Phase ON, 0–52 points	7
MDS - UPDRS III	Phase ON, 0–132 points	14
Item 3.10	Gait	1
Item 3.11	Freezing of Gait	0
Item 3.12	Postural Instability	1
MDS - UPDRS IV	Phase ON, 0–24 points	0
Neuropsychological examination	MMSE (0–30)	28
	MoCA (0–30)	26
	BDI-II	11
Laboratory Tests –Complete Blood Count (CBC)	White Blood Cells (WBC)	4.38 × 10^3^/µL
	Red Blood Cells (RBC)	4.24 × 10^6^/µL
	Hemoglobin (Hb)	13.50 g/dL
	Hematocrit (Hct)	42%
	Mean Corpuscular Volume (MCV)	99.0 fL
	Mean Corpuscular Hemoglobin (MCH)	31.8 pg
	Mean Corpuscular Hemoglobin Concentration (MCHC)	32.1 g/dL
	Red Cell Distribution Width (RDW)	12.5%
	Platelet Count	185 × 10^3^/µL
	Mean Platelet Volume (MPV)	950fL

BMI, body mass index; H&Y, Hoehn and Yahr; LEDD, levodopa equivalent daily dose; UPDRS, Unified Parkinson's Disease Rating Scale; MMSE, mini-mental state examination; MoCA, montreal cognitive assessment; BDI-II, Beck Depression Inventory – Second Edition.

Hematochemical parameters were within normal ranges, and no orthostatic hypotension was detected according to current clinical criteria (27). No cognitive impairment was observed. Clinical evaluation was performed in ON medication state, indicated mild disease severity, consistent with Hoehn and Yahr stage 2, with no evidence of freezing of gait or postural instability.

Disease severity was classified as slight based on the Movement Disorder Society–Unified Parkinson's Disease Rating Scale (MDS-UPDRS), detailed scores are reported in Table 1. No motor fluctuations or dyskinesias were present. No clinical, neurological, or biochemical contraindications to participation in structured physical exercise were identified.

Given the patient’s eligibility and physical condition, and in accordance with routine clinical decision-making within the rehabilitation program, the participant took part in the intensive aerobic training pathway foreseen by the METEX-PD study, conducted in accordance with the Declaration of Helsinki.

### 2.2 Measures and instruments

Prior to initiating the exercise training, all participants underwent a comprehensive clinical and functional evaluation. These assessments were conducted according to the methodology previously described (12), and included:

Functional-motor assessments
  • MDS-UPDRS: Parts I (non-motor aspects of experiences of daily living), II (motor aspects of daily living), III (motor examination), and IV (motor complications) were administered to assess motor symptoms and treatment-related complications.
  • Six-Minute Walking Test (6MWT): Evaluated functional exercise capacity, reported as distance walked in meters.
Clinical and Neuropsychological Assessments
  Cognitive and non-motor domains were evaluated using a battery of validated clinical-functional scales. Specifically:
  • Cognitive function was assessed through the administration of MMSE and MoCA (25, 26).
  • Depressive symptoms were evaluated using the BDI-II, a 21-item self-report inventory widely used to assess the severity of depressive symptomatology in clinical populations (28).
Laboratory

Laboratory analyses were performed according to routine clinical practice.

## 2 Materials and methods

Prior to entering the intensive aerobic training program, the participant completed a familiarization phase to ensure safety, adaptation to equipment, and accurate calibration of exercise intensity, trough an incremental submaximal exercise. This phase included two components: treadmill and metabolic equipment familiarization.

Treadmill familiarization: the participant first completed a walking session at a constant speed of 3.6 km/h. This pace was selected based on previous literature identifying it as a low-intensity, low-energy-expenditure walking speed in healthy older adults (29). The aim of this session was to allow participants to acclimate to treadmill use in a controlled and comfortable setting.

Metabolimeter (K5) familiarization: During a dedicated session, the participant was introduced to the use of the K5 portable metabolimeter (COSMED, Rome, Italy). After donning the face mask and confirming proper fit and signal stability, they began walking at 3.6 km/h for a 3-minute warm-up (WU) phase. Subsequently, the walking speed was increased to reach a metabolic workload corresponding to the target range for vigorous-intensity aerobic exercise (6.0–8.8 METs). Oxygen uptake ($\dot{V}$O₂), heart rate (HR), and ventilatory parameters were continuously monitored. To ensure participants remained below the second ventilatory threshold (VT2) and to prevent excessive metabolic stress, capillary blood lactate samples were collected by a sports medicine physician. Exercise intensity was adjusted to maintain blood lactate concentration below 4 mmol/L (30).

Based on this assessment, the treadmill speed for the training sessions was individually set at 5.0 km/h, which met the intensity criteria without exceeding the physiological safety thresholds. The slope was set at 1% to simulate level overground walking rather than belt-driven treadmill walking (31). Following 45 minutes of exercise (EXE), the participant performed a 5-minute cool-down (CD) phase at the same speed used during the warm-up (WU).

The training protocol consisted of 20 treadmill walking sessions over a 4-week period (5 sessions per week). It included a pre-test to determine the workload to be maintained throughout the training period, a mid-test conducted after the 10th session, and a post-training test to evaluate the participant's physiological adaptation to the workload performed. Following the ACSM guidelines for aerobic exercise prescription (32), as well as recommendations from the scientific literature on aerobic training in PD (14, 33), the activity was designed to be of “high intensity”, corresponding to an energy expenditure between 6.0 and 8.8 METs. The safety of exercising at this intensity range in individuals with PD has been previously established in the literature (34), supporting its feasibility and tolerability in clinical training settings, under the supervision of exercise professionals and qualified healthcare specialists.

A progressive increase in workload was planned, based on the subject's physiological responses, in order to maintain the target intensity parameters throughout all training sessions. Each session included 3 minutes of WU, 45 minutes of constant-load aerobic exercise (EXE), and 5 minutes of CD. During the three test sessions, the K5 portable metabolic analyzer was used to directly detect O2 consumption and monitor HR. Additionally, a sports medicine physician assessed capillary blood lactate concentration sampling via earlobe to ensure that the ventilatory threshold was not exceeded, thereby confirming that the exercise intensity was sustainable for the planned duration. Sampling was performed at the halfway point of the exercise and at the 1^(st), 3^(rd) and 5^(th) minute of the recovery period.

In all training sessions, HR was continuously monitored using the H10 Heart Rate Sensor (© Polar Electro, 2025). Following a familiarization session with the K5 system, a constant treadmill speed of 5 km/h was set for the 45-minute EXE phase in the first 10 sessions. After this initial training block, a retest was performed using the K5 system and lactate measurements sampling to monitor the training adaptations. Based on these results, the workload was adjusted to 5.5 km/h for the final 10 sessions. The WU and CD speeds remained unchanged throughout the intensive-exercise rehabilitation program.

### 2.1 Statistical analysis

Statistical analyses were performed using GraphPad Prism (GraphPad Software, San Diego, CA, USA). Data normality was assessed using the Shapiro–Wilk test. Continuous variables are presented as mean ± standard deviation (SD). Differences across conditions were analyzed using a one-way repeated-measures analysis of variance (ANOVA). When a significant main effect was detected, *post-hoc* comparisons were performed using Tukey's correction. The level of statistical significance was set at *p* < 0.05.

## 3 Results

The patient successfully completed the entire aerobic exercise program without interruptions or adverse events. The workload intensity achieved during training sessions, corresponding to a walking speed of 5.0 km/h (first 2 weeks) −5.5 km/h (last two weeks). Post-exercise blood lactate concentrations consistently remained below 2 mmol/L during the recovery phase (Figures 1A,D).

**Figure 1:**
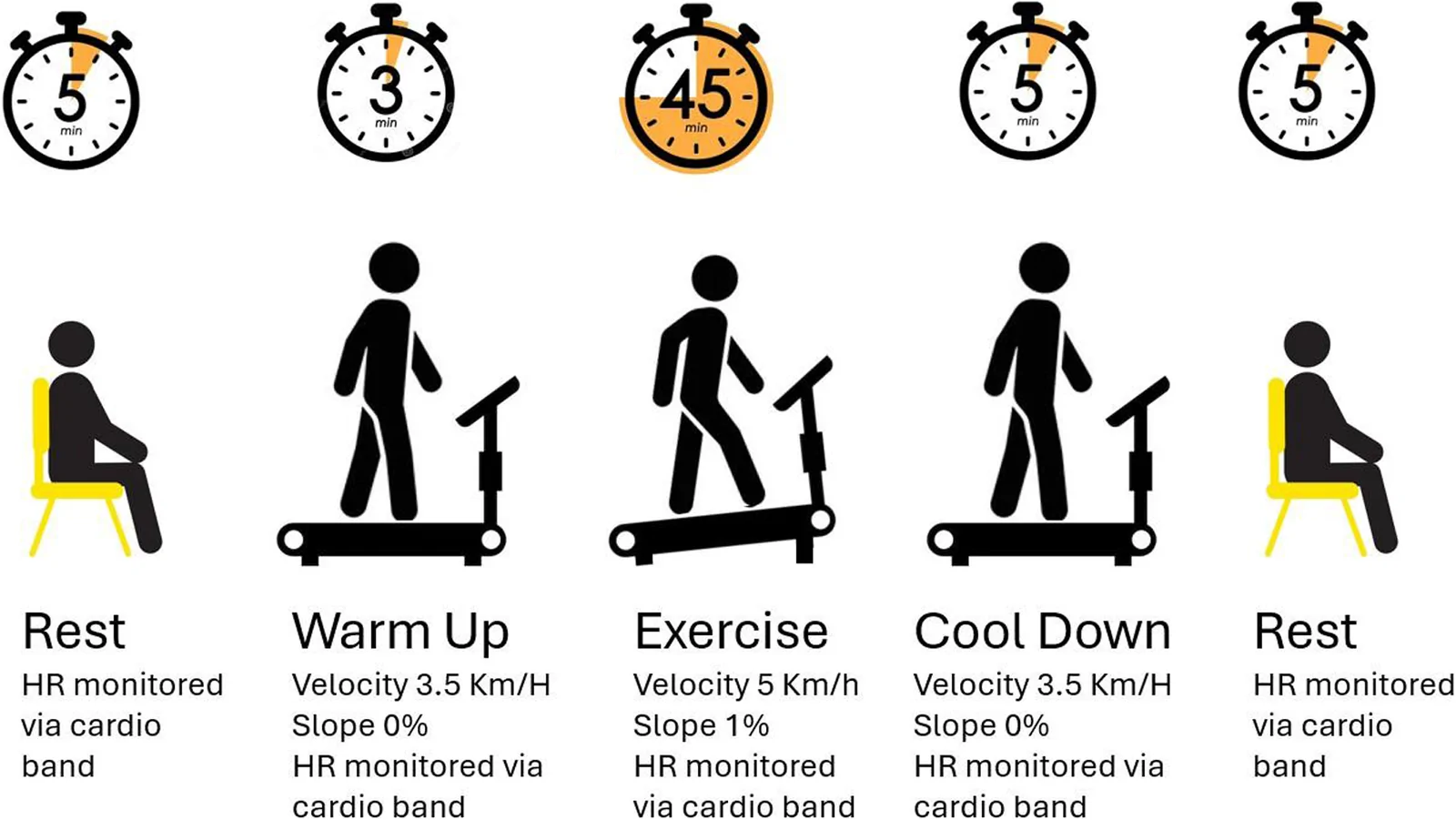
Description of intensive-exercise rehabilitation program. Aerobic treadmill training protocol comprising seated rest, warm-up, exercise, and cool-down phases. Training was performed at 3.5 km/h (0% slope) during warm-up and cool-down and at 5.0 km/h (1% slope) during the exercise phase, with continuous HR monitoring via cardio band. HR, Heart Rate.

At the 6-Minute Walking Test, gait speed, cadence, and step length values were above normative ranges at both pre- and post-treatment assessments. No clinically meaningful changes in overall walking performance were observed between evaluations, with preservation of spatiotemporal gait parameters and a slight increase in the global symmetry index, indicating a stable functional walking capacity over the observation period.

All testing sessions were supervised to ensure patient safety and adherence to the procedures established within the rehabilitation program. Exercise workload was kept constant across all testing sessions in order to physiologically evaluate the adaptations induced by the training intervention under standardized exercise conditions. Mean MET values recorded during the three monitored sessions using the K5 metabolic analyzer are presented in Figure 2A, while heart rate (HR), oxygen uptake ($\dot{V}$O₂), and blood lactate responses are shown in Figures 2B–D, respectively.

**Figure 2:**
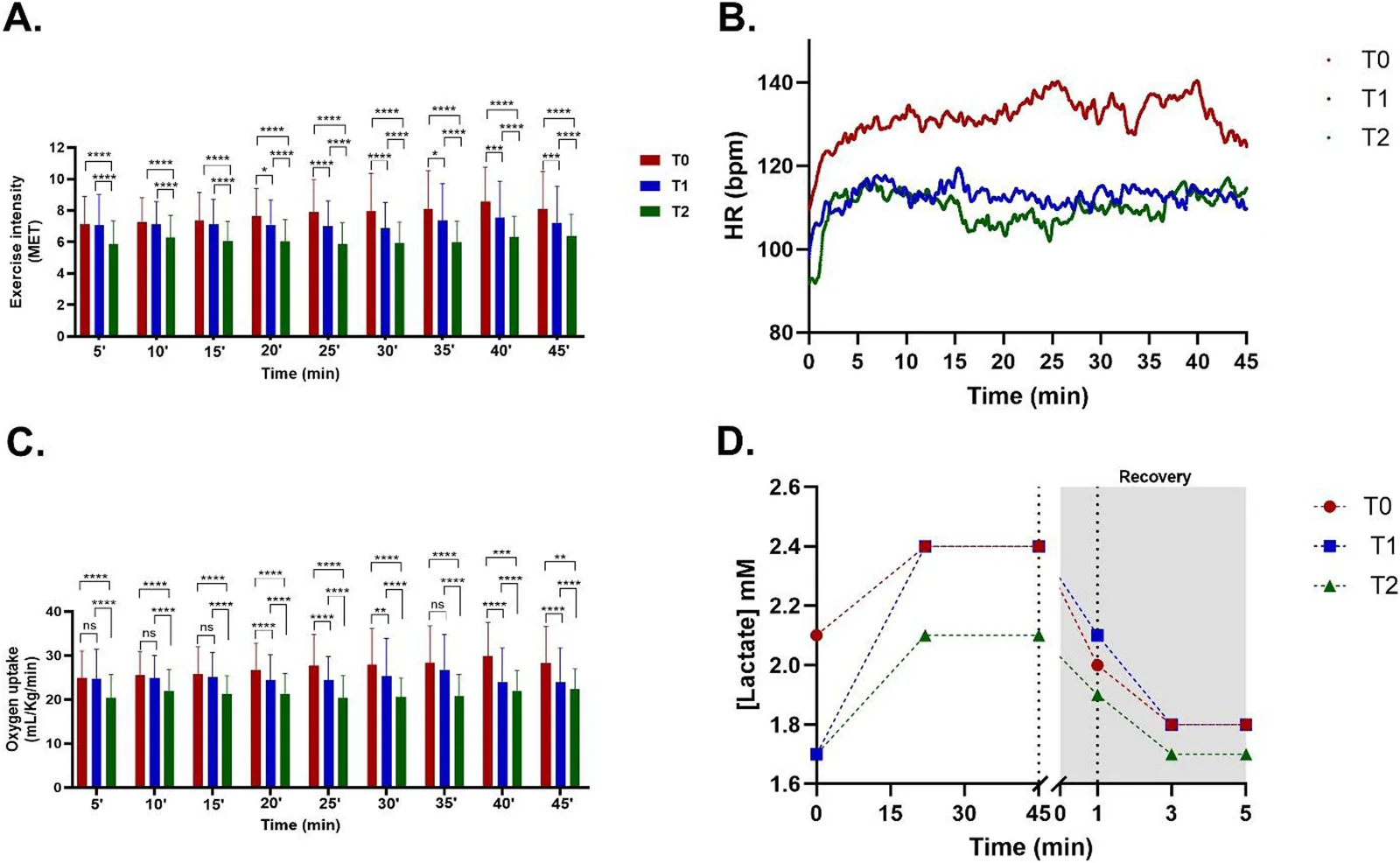
Metabolic adaptations of a patient with PD to an intensive aerobic rehabilitation program. MET = Metabolic Equivalent of Task; HR = Heart Rate; bpm = beats per minute; R = Heart Rate; **(A)** Exercise intensity expressed as METs, **(B)** HR in bpm, and **(C)** oxygen uptake ($\dot{V}$O₂), expressed as mL·kg⁻^1^·min⁻^1^, were evaluated at three time points (T0 – baseline, T1 – mid-program, and T2 – end of training). For exercise intensity, HR, and oxygen uptake (VO₂), values are presented as smoothed mean curves to reduce short-term fluctuations. Data collected during each 45-min exercise session were compared across 5-minute blocks at T0, T1, and T2. Statistical analyses were performed using GraphPad Prism 10, applying a one-way repeated-measures ANOVA followed by Tukey's *post-hoc* test, in accordance with GraphPad conventions (*p < 0.05, **p < 0.01, ***p < 0.001, ****p < 0.0001).

In accordance with the METEX-PD trial protocol (12), which defines the target weekly exercise volume as 1,350–1,980 METs•min/week, the patient successfully achieved the expected range, with a volume evaluated at different time points T0: 1752.2 ± 466.1; T1: 1612.7 ± 432.1; T2: 1369.2 ± 307.6 METs•min/week. Consequently, a substantial progressive metabolic adaptation was observed from the initial measurement (T0) to the conclusion of the training period (T2). This adaptation was also reflected by reductions in heart rate (HR) and $\dot{V}$O₂ during the 45-minute exercise sessions, and recovery blood lactate levels indicating enhanced aerobic efficiency at a constant workload (see Figure 2).

As demonstrated in Table 2, the energy cost and energy expenditure measured during 45 minutes of aerobic exercise confirmed the metabolic adaptation, with a decrease of 9.06% at T1 and 20.45% at T2 with respect to T0.

**Table 2:** Comparison of theoretical and measured METs during treadmill walking in an older adult With Parkinson's disease.

Time point	Velocity (km/h)	METs – Theoretical (Compendium 2024, elderly)	METs – Measured (PD patient) (mean ± SD)	Energy cost (Joule/kg/m)	Total Energy expenditure (KJoule)
T0	5.0	5.3	7.8 ± 2.1	6.8	25.4
T1	5.0	5.3	7.2 ± 1.9	6.2	23.1
T2	5.0	5.3	6.1 ± 1.4	5.4	20.2

MET, metabolic equivalent of task; SD, standard deviation.

Over the entire 4-week training period, HR values showed a progressive decrease from session 1 to session 20 (Figure 2B). From the HR data at T0 and T1 (Figures 3B,C), a more stable physiological response to the exercise workload appears to be present. In contrast, at T2 a reduction in HR is observed starting from approximately the 10th minute, followed by a change in slope around the 30th minute). The analysis of HR data collected during the training sessions revealed a consistent intra-session trend during the 45-minute exercise bouts, suggesting a similar overall pattern of HR behaviour was already present throughout the training period, including prior to T1 (Figure 4).

**Figure 3:**
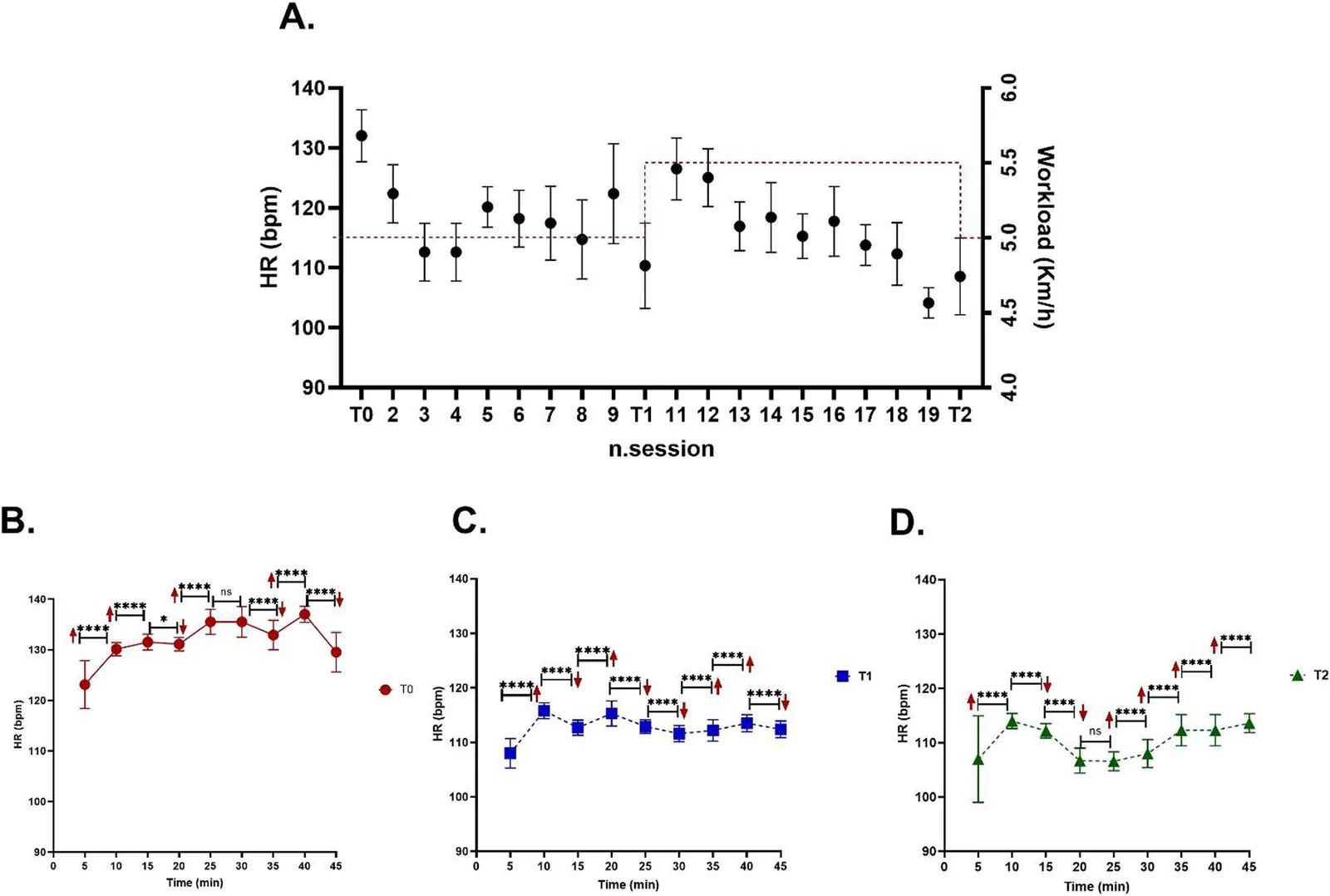
Heart rate during aerobic exercise sessions in a patient With PD. HR, Heart Rate; n, Number; SD, Standard Deviation. HR Mean ± SD recorded across 20 aerobic exercise sessions. **(B–D)** Intrasession HR variability at three timepoints (T0, T1, T2). For this analysis, each 45-min exercise session was segmented into consecutive 5-minute blocks, and within each timepoint, each block was compared with the following one to assess short-term fluctuations. Statistical analyses were performed in GraphPad Prism 10 using a one-way repeated-measures ANOVA followed by Tukey's *post-hoc* test. Significance thresholds follow GraphPad conventions: (*p < 0.05, **p < 0.01, ***p < 0.001, ****p < 0.0001; ns = not significant).

**Figure 4:**
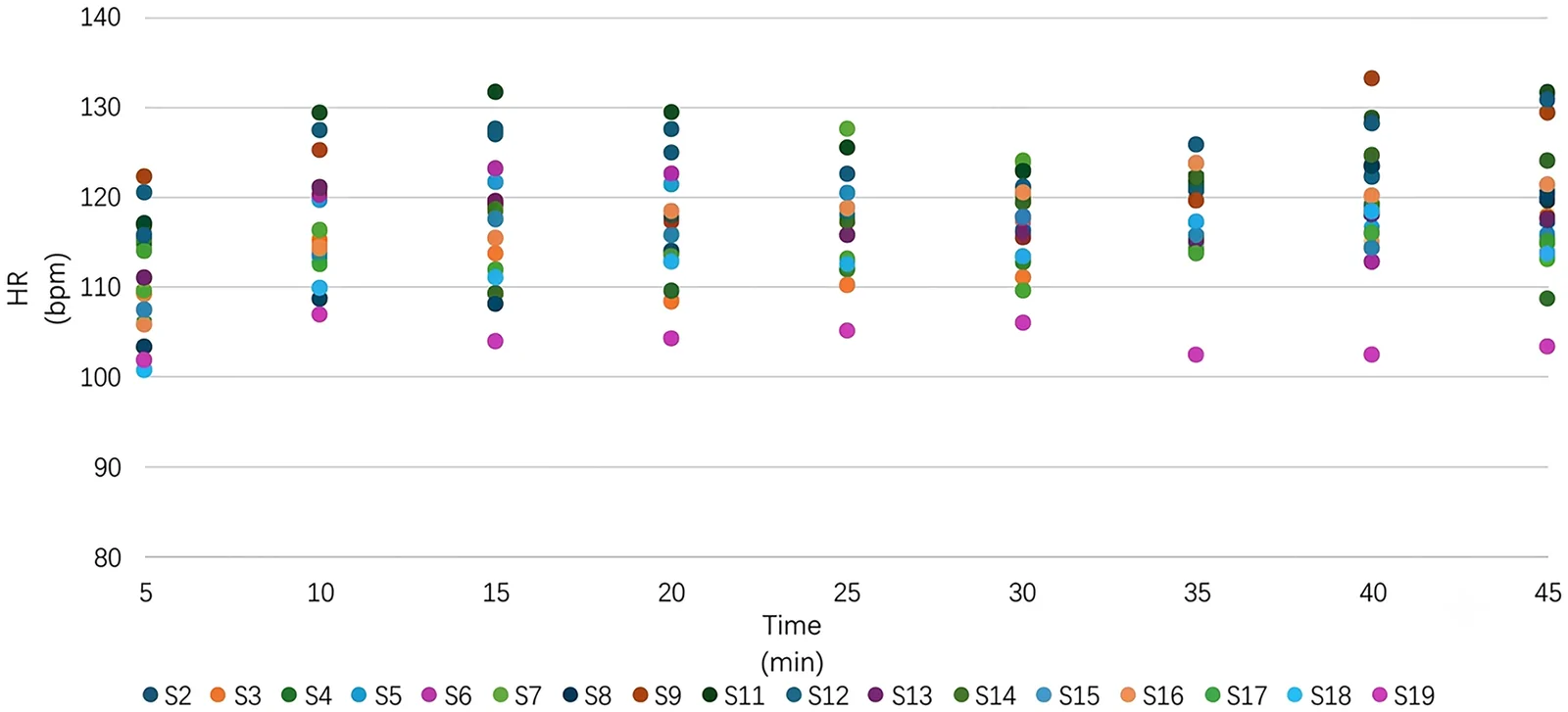
HR values for all training sessions.

Considering the HR accuracy, with less than 4% error in monitoring performance compared with Holter evaluation (35), we can reasonably exclude that these findings are attributable to measurement variability.

In addition to the aforementioned adaptations, a change in respiratory exchange ratio (RER) was observed across the monitored sessions (Figure 5). Specifically, RER values were higher at T2 compared with T0 and T1. This increase appears to be primarily attributable to a higher respiratory frequency (RF) recorded at T2, rather than to an increase in metabolic stress. Indeed, despite the elevated RER, exercise intensity and workload remained constant, and blood lactate concentrations continued to be around 2 mmol/L (Figure 2D), supporting the predominance of aerobic metabolism. The higher ventilatory response at T2 may therefore reflect an altered ventilatory strategy or an adaptive respiratory pattern during prolonged exercise, rather than a shift toward greater anaerobic contribution.

**Figure 5:**
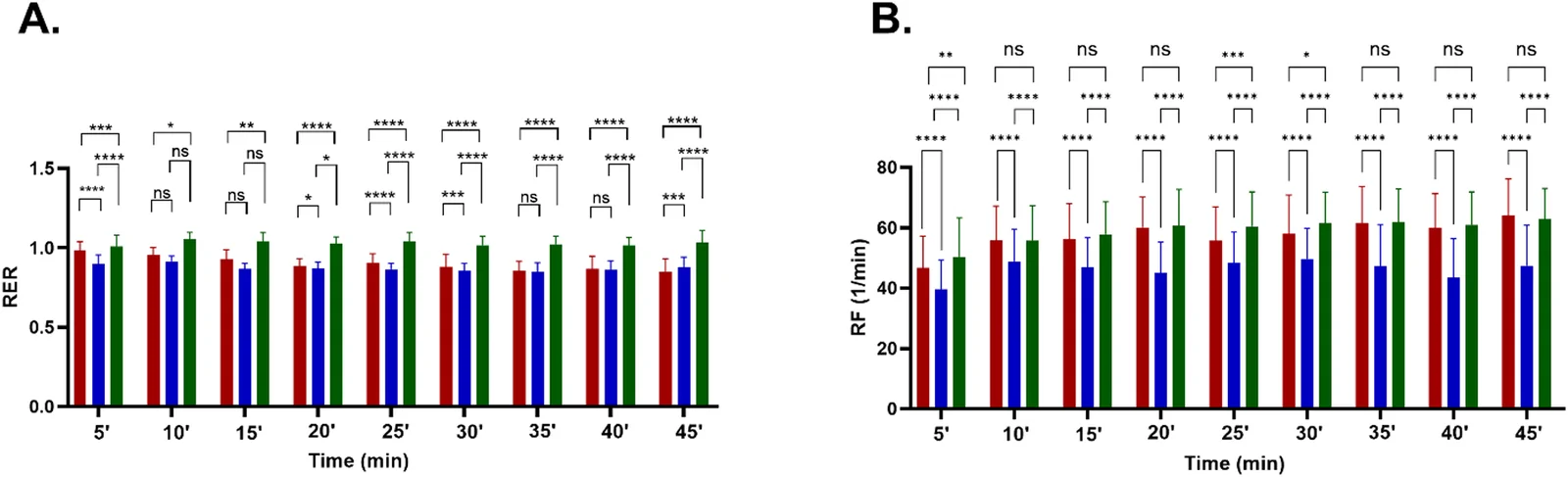
Respiratory Parameters During Aerobic Exercise Sessions in a Patient With PD. RER, respiratory exchange ratio; RF, respiratory frequency; SD, standard deviation. **(A)** RER Mean ± SD recorded across 20 aerobic exercise sessions at three timepoints (T0, T1, T2). **(B)** RF Mean ± SD measured during the same sessions and timepoints. For both analyses, each 45-min exercise session was segmented into consecutive 5-minute blocks, and within each timepoint, each block was compared with the following one to assess short-term fluctuations. Statistical analyses were performed in GraphPad Prism 10 using a one-way repeated-measures ANOVA followed by Tukey's *post-hoc* test. Significance thresholds follow GraphPad conventions: (*p < 0.05, **p < 0.01, ***p < 0.001, ****p < 0.0001; ns = not significant).

Considering this exercise workload, the acute and chronic responses were evaluated for insulin-like growth factor 1 (IGF-1) according to METEX-PD study protocol, data are shown in Table 3.

**Table 3:** Chronic vs. Acute Exercise IGF1 response.

Acute exercise					
First session – Acute response			Last session- Acute response post-training		
IGF-1 (µg/L) Pre-training	IGF-1 (µg/L) Post-training	*Δ*IGF-1 (µg/L)	IGF-1 (µg/L) Pre-training	IGF-1 (µg/L) Post-training	*Δ*IGF-1 (µg/L)
97	101	4	106	106	0

**Table:** 

Chronic Exercise		
Baseline (pre-first session) IGF-1 (µg/L)	End-of-training (Pre-last session) IGF-1 (µg/L)	*Δ*IGF-1 (µg/L)
97	106	9

IGF-1, Insulin-Like Growth Factor 1; *Δ*IGF-1, Difference of IGF-1 between pre and post training.

The acute responses observed in the serum IGF-1 levels were minimal and fell within the anticipated range of biological variability. This finding is consistent with the known kinetics of IGF-1 synthesis and release, which is primarily hepatic and does not typically respond rapidly to short-term stimuli. In contrast, following the conclusion of the 20-session training program, which corresponded to a weekly exercise volume and intensity of approximately T0: 1752.2 ± 466.1, T1: 1612.7 ± 432.1, T2: 1369.2 ± 307.6 METs•min/week, a significant increase in IGF-1 levels was observed. This finding is consistent with the extant literature, which demonstrates that chronic aerobic exercise in healthy individuals, when performed with sufficient training volume, can elicit a long-term upregulation of the IGF-1 system (36). The observed rise in IGF-1 therefore reflects a biological adaptation to repeated exercise exposure rather than an acute response.

### 3.1 Follow-up and outcomes

The patient completed all 20 training sessions scheduled under the protocol and tolerated the intervention well. The primary aim of this case report was to characterize the patient's peculiar behavioral profile rather than to assess treatment efficacy or clinical outcomes. Therefore, standardized clinician- or patient-reported outcome measures were not collected. During follow-up, the patient's motor performance remained stable after the training period, without evidence of clinically relevant deterioration or unexpected adverse events. No additional diagnostic findings or relevant changes in clinical status were observed during the observation period.

### 3.2 Patient perspective

The participant reported a positive experience throughout the intervention and expressed high motivation to adhere to the exercise program. No adverse events or exercise-related complications occurred during the study period, and all scheduled training sessions were completed without interruption, reflecting excellent adherence. The participant perceived progressive improvements in physical capacity and exercise tolerance throughout the intervention, which further reinforced motivation to continue exercising. At the end of the training period, the participant expressed overall satisfaction with the intervention and voluntarily maintained an active lifestyle by continuing to perform regular physical exercise independently at home after completion of the supervised training period.

## 4 Discussion

In this work, cardiovascular responses were evaluated through heart rate monitoring, while exercise intensity was assessed via gas exchange analysis and blood lactate concentration during constant-load aerobic exercise in an individual with PD. This study highlights the effects of aerobic exercise on heart rate and oxygen consumption, which, with same load, decrease progressively from T0 to T2. This is also confirmed by the lactate concentration, which follows the same trend, as reported in Figure 1D.

These results indicate an improvement in cardiovascular efficiency and aerobic metabolism. We can hypothesize that the minor lactate concentration may be related to more fluid walking biomechanics, thereby facilitating better synchronization between agonistic and antagonistic muscles during the walking cycle. These outcomes support the growing body of evidence indicating that aerobic exercise represents a valuable adjunctive therapy in PD management. In particular, the improvements observed in aerobic capacity, showed by the reduction in energy expenditure at the same workload, are consistent with findings from clinical trials literature reporting benefits in motor control. A more detailed analysis of HR revealed a consistent pattern across exercise sessions. Specifically, HR slightly declined by about 3% between the 10th and 30th minute of exercise and subsequently increased about 4% toward the end of the session, despite the workload remaining constant.

In fact, as we can see from Figures 1, 2B–D, at the beginning of exercise, HR rises rapidly in response to the central and peripheral adaptation with respect to the imposed workload. However, despite the constant load during testing and training, a decrease in HR can be observed in every test trial around the 30th minute training sessions. We hypothesize that this pattern may reflect the CI (20, 37). This condition is directly linked to the key role that dopamine plays in the regulation of autonomic cardiovascular control, as it influences catecholaminergic pathways, that stimulate HR. For example, in PD, the reduction of dopaminergic activity may contribute to a lower chronotropic response during exercise (38). Moreover, it's important to stress that CI is associated with autonomic dysfunction, which is also associated with fatigue (39), common causes of dropout in training programs for people with PD (40). CI has already been described in PD, but more findings are needed to better comprehend its physiological mechanisms in PD patients (19, 41), not only referring to cardiac but also to respiratory regulation (42).

In this regard, despite stable metabolic demands, cardiorespiratory anomalies surfaced during the extended phase of constant-load walking.

Respiratory frequency rose by +3% breaths·min⁻^1^ leading to a concomitant increase in RER. Crucially, this occurred without any corresponding rise in metabolic VO_2_, VCO_2_ production from muscular work, or METs, indicating non-metabolic hyperpnea.

This cardiorespiratory dissociation suggests a central blunting of ventilatory control (43). We can hypothesize that this phenomenon could be linked to CI or the distinctive cardiac response of the subject. Both abnormalities may reflect a shared pathophysiological substrate: central brainstem autonomic dysfunction and a disrupted sympathetic/parasympathetic balance, which decouples peripheral muscular demands from central cardiorespiratory reflexes.

During the final phase of exercise, heart rate (HR) increased despite a constant workload, a phenomenon that may be explained by CVD (21).

Most PD exercise protocols rely on HR or heart rate reserve (HRR) to prescribe workload, particularly when exercise intensity is established through maximal cardiopulmonary exercise testing (CPET), which remains the gold standard for determining relative exercise intensity. However, in clinical and real-world rehabilitation settings where CPET may not be routinely available, HR-derived parameters may be influenced by physiological phenomena such as CVD and CI. Under these circumstances, exercise intensity may not always accurately reflect the underlying metabolic demand, potentially leading to over- or underestimation of the actual workload. Specifically CVD may progressively increase HR despite constant workload, whereas CI may blunt the HR response to exercise. These factors could contribute to variability in exercise tolerance, adherence, and physiological adaptation, particularly in individuals already experiencing fatigue or reduced exercise tolerance.

Although the present findings are limited by the single-case design and cannot be generalized to the PD population, they provide preliminary insights that may inform future research. Specifically, the observed physiological response suggests that heart rate-based prescription alone may not fully capture exercise intensity adaptations during prolonged continuous exercise and not only in graded exercise (41). Future studies should investigate whether external workload parameters and other physiological markers may complement HR monitoring to optimize individualized exercise prescription in PD (44). This perspective is similar to the approach adopted in endurance sports, where CVD induced by prolonged exercise can reduce the reliability of heart rate as the sole indicator of exercise intensity (45, 46), leading to the strategy of always combining data of external load (power, speed, duration and pace) and internal load (HR, HRR, perceived exertion) (47–49) to guide the monitoring and prescription of training.

So, given that perceived exertion may be influenced by disease-related motor, cognitive, and psychological factors in individuals with PD (3, 20, 39, 50), objective physiological parameters, such as gas exchange and blood lactate measurements, may provide potentially less confounding information for a more accurate assessment of exercise intensity in this population (44, 51).

A further finding concerns the MET values achieved during exercise. Although the subject was physically active prior to the intervention, the measured MET value was higher than the one reported in the Compendium of physical activity (29), referring to healthy older adults walking at the same treadmill speed (T0 + 47%; T1 + 35,8%; T2 + 15%) and referring to other categories of walking impairments (52), confirming what has already been reported in literature (53, 54). Altered muscle activation patterns, rigidity, bradykinesia, and reduced mechanical efficiency may substantially increase energy expenditure and contribute to the early onset of fatigue. In this work, the results suggest improved physiological efficiency in terms of cardiovascular (HR percentage difference T0, T2= −16,5%) and metabolic demand (MET Percentage difference T0, T2= −19,6%). However, not accounting for variability in MET values in people with PD may result in an underestimation of their internal workload, potentially leading to less appropriate exercise prescription.

HR trend across the test trials also changed with training. At T0 and T1, HR values were generally higher and relatively stable throughout the test. Conversely, at T2, HR was lower during the middle phase of exercise and increased progressively during the final minutes, revealing a possible CV drift. This pattern could be consistent with an improved stroke volume at exercise onset due to training-induced cardiovascular adaptations, together with a late drift driven by progressive thermal load, fluid redistribution, and induced peripheral fatigue during prolonged exercise (55).

Interestingly, analysis of HR recordings collected during the training sessions suggested that this biphasic HR behaviour, characterized by an initial reduction followed by a progressive late increase, was present even before T2 and appeared more evident outside the standardized testing environment. One possible explanation is that the controlled testing setting may have partially masked these dynamics through anticipatory sympathetic activation and psychophysiological stress responses, leading to higher and more stable HR values during the initial phases of exercise. In contrast, HR behaviour recorded during routine training sessions may better reflect the subject's spontaneous physiological adaptation to prolonged constant-load exercise.

At T2, the late phase of exercise was also characterized by a concomitant increase RF (Figure 5 panel B) without a parallel rise in $\dot{V}$O₂ (Figure 2 panel C), suggesting the presence of relative hyperventilation. This response may reflect a late sympathetic activation during prolonged effort, which could be amplified by the autonomic dysfunction commonly observed in Parkinson's disease (55).

In addition, sustained stimulation of limb muscle during prolonged walking may enhance ventilatory drive through afferent inputs to the respiratory centers (56), contributing to the dissociation between RF, $\dot{V}$CO2 and $\dot{V}$O2. In fact, the RER values are altered and, when considered in isolation, could suggest a greater contribution of anaerobic metabolism compared to aerobic metabolism. However, the subject's metabolic demand remains predominantly aerobic, as confirmed by the blood lactate concentration and the subject's ability to walk at a steady pace.

The increase in respiratory frequency has relevant implications for fluid balance. Hyperventilation augments perspiratio insensibilis, namely the continuous loss of water through the respiratory tract and skin that occurs independently of sweating and raises with the increase in exercise intensity. During prolonged aerobic exercise, the combination of increased ventilation, sweating, and elevated environmental or body temperature can substantially increase total water loss and promote transient reductions in plasma volume during effort (57), as seen in T0. Although these losses may be temporarily masked in the post-exercise period by interstitial-to-intravascular fluid shifts and cool-down–induced hemodilution (58). These features likely contribute to the late CV drift observed at T2 and represent an important factor to consider during exercise prescription in PD.

Post-exercise plasma volume variation (*Δ*PV), estimated using the Dill and Costill equation (59), was negative at T0 and T1, consistent with acute hemoconcentration due to fluid loss during exercise. In contrast, *Δ*PV was positive at T2. This shift suggests the coexistence of two physiological mechanisms: a rapid post-exercise return of fluids from the interstitial to the intravascular compartment, favored by the 5-minute low-intensity cool-down (60), and a likely chronic plasma volume expansion induced by adaptation to endurance training. Therefore, exercise induced a key central adaptation to aerobic training, improving venous return, a stroke volume and contributing to the lower HR as observed at T2 (61).

These findings confirm that with consistent physical activity, PD patients can achieve improvements in functional capacity and cardiorespiratory fitness similar to healthy adults, thus endurance exercise effects in PD patients are similar to healthy elderly (62, 63).

However, the coexistence of elevated MET values, late CV drift, and relative hyperventilation highlights the complex interaction between mechanical inefficiency, autonomic regulation, and fluid balance in PD. Our results highlight the importance of individualized, instrument-based monitoring for safe and effective exercise prescription in this population.

In our experimental study which involved 4 weeks of standardized and quantified aerobic training, we observed a change in IGF-1 levels post-intervention, consistent with a potential modulatory effect of chronic exercise on this neurotrophic factor (33).

In animal models of PD, exercise-induced increases in IGF-1 have been shown to correlate with neuroplastic phenomena, including synaptogenesis, synaptic remodeling, and activation of neurotrophic signaling pathways (7, 33, 64–67).

However, in humans, despite evidence suggesting that aerobic exercise may modulate circulating IGF-1 levels, definitive data establishing a dose–response relationship between quantified aerobic exercise, changes in plasma IGF-1, and direct effects on neuroplastic processes in Parkinson’s disease are still lacking (51).

These findings may help stimulate further research aimed at exploring the relationships between altered physiological mechanisms in PD. It is also hoped that future clinical trials, similar in design but including age-matched healthy participants, will help to better contextualise the observed metabolic equivalent (MET) values and cardiorespiratory responses.

Nevertheless, if our interpretation of this case study is correct and there is the manifestation both of CI and CVD, it would suggest that, even in the presence of impaired dopaminergic modulation, compensatory mechanisms related to thermoregulation can override the reduced cardiac activation, leading to a secondary increase in HR to maintain physiological stability during exercise. As for hyperventilation, it is a phenomenon that warrants further study, including its regard to its association with CI.

Although limited by its single-patient design, this report provides a comprehensive physiological characterization of the response to standardized high-intensity aerobic exercise in Parkinson's disease. The simultaneous assessment of metabolic efficiency, heart-rate behaviour, blood lactate, ventilatory responses and circulating IGF-1 offers mechanistic insights into exercise adjustments and adaptations as first results of the METEX-PD study protocol and may contribute to the monitoring and optimization of individualized exercise prescription in future clinical studies.

In conclusion, when dealing with subjects with PD, it would be useful and necessary to take into account all parameters, such as $\dot{V}$O_2_, MET, RF and blood lactate concentration. Furthermore, a better comprehension of the autonomic response to exercise in PD and the physiological mechanism behind it, could provide more information when proposing a training protocol. Such an approach would contribute to the development of truly individualized exercise prescriptions for people with PD, reinforcing the notion that “exercise is medicine”.

## Data Availability

The datasets presented in this article are not readily available because the dataset contains clinical and physiological data from a single participant. Public sharing is restricted to protect participant confidentiality and privacy. Requests to access the datasets should be directed to a.rodio@unicas.it.

## Supplementary material

Supplementary Data Sheet 1 CARE checklist.
