# Supplementary material for: Case Report: Metabolic adaptations to intensive aerobic training in Parkinson's disease: autonomic and physiopathological response
Source: Front Sports Act Living. 2026 Oct 7;8:1920699. doi: 10.3389/fspor.2026.1920699 (PMC13683664; doi:10.3389/fspor.2026.1920699)
Supplement: Supplementary file 1 [file Datasheet1.pdf]

CARE case report guidelines

CARE Checklist of information to include when writing a case report

(cc) BY-NC-ND

Topic	Item	Checklist item description	Reported on Line
Title	1	The diagnosis or intervention of primary focus followed by the words "case report"	Title
Key Words	2	2 to 5 key words that identify diagnoses or interventions in this case report, including "case report"	key words section
Abstract (no references)	3a	Introduction: What is unique about this case and what does it add to the scientific literature?	Introduction
	3b	Main symptoms and/or important clinical findings	Table 1 and 1.2 section
	3c	The main diagnoses, therapeutic interventions, and outcomes	Introduction
	3d	Conclusion—What is the main "take-away" lesson(s) from this case?	Introduction
Introduction	4	One or two paragraphs summarizing why this case is unique (**may include** references)	1, 3 and 4 section
Patient Information	5a	De-identified patient specific information	1.2 section
	5b	Primary concerns and symptoms of the patient	1.2 section
	5c	Medical, family, and psycho-social history including relevant genetic information	1.2 section
	5d	Relevant past interventions with outcomes	1.2 section
Clinical Findings	6	Describe significant physical examination (PE) and important clinical findings	3 section
Timeline	7	Historical and current information from this episode of care organized as a timeline	2 section
Diagnostic Assessment	8a	Diagnostic testing (such as PE, laboratory testing, imaging, surveys).	Table 1
	8b	Diagnostic challenges (such as access to testing, financial, or cultural)	1.2 section
	8c	Diagnosis (including other diagnoses considered)	1 and 1.2 section
	8d	Prognosis (such as staging in oncology) where applicable	not applicable
Therapeutic Intervention	9a	Types of therapeutic intervention (such as pharmacologic, surgical, preventive, self-care)	1.3, 2 sections
	9b	Administration of therapeutic intervention (such as dosage, strength, duration)	1.3 and 2 sections
	9c	Changes in therapeutic intervention (with rationale)	table 1, 1.2, 1.3, 2 sections
Follow-up and Outcomes	10a	Clinician and patient-assessed outcomes (if available)	3.1 section
	10b	Important follow-up diagnostic and other test results	3.1 section
	10c	Intervention adherence and tolerability (How was this assessed?)	3.1 section
	10d	Adverse and unanticipated events	3.1 and 3.2 sections
Discussion	11a	A scientific discussion of the strengths AND limitations associated with this case report	discussion section
	11b	Discussion of the relevant medical literature **with references**	discussion section
	11c	The scientific rationale for any conclusions (including assessment of possible causes)	discussion section
	11d	The primary "take-away" lessons of this case report (without references) in a one paragraph conclusion	discussion section
Patient Perspective	12	The patient should share their perspective in one to two paragraphs on the treatment(s) they received	3.2 section
Informed Consent	13	Did the patient give informed consent? Please provide if requested	Yes ☒ No ☐
